# Serous Otitis Media Secondary to a Middle Ear Neuroendocrine Tumor of Five-Year Duration

**DOI:** 10.7759/cureus.110910

**Published:** 2026-06-15

**Authors:** Jose Rodrigo Alcantara Dzib, Jose Carlos Rivera Caceres, Patricia Berenice Bolado García, Alondra Karent Balbuena Coutiño, Rolando Adan Medina Pinto

**Affiliations:** 1 Department of Pathology, Unidad Médica de Alta Especialidad, Hospital de Especialidades Centro Médico Nacional “Ignacio García Téllez”, Instituto Mexicano del Seguro Social, Mérida, MEX; 2 Department of Otorhinolaryngology, Unidad Médica de Alta Especialidad, Hospital de Especialidades Centro Médico Nacional “Ignacio García Téllez”, Instituto Mexicano del Seguro Social, Mérida, MEX; 3 Department of Health Research, Unidad Médica de Alta Especialidad, Hospital de Especialidades Centro Médico Nacional “Ignacio García Téllez”, Instituto Mexicano del Seguro Social, Mérida, MEX

**Keywords:** immunohistochemistry staining, neuroendocrine tumor, otitis media with effusion, otolaryngology, pathology

## Abstract

Neuroendocrine tumors (NETs) are extremely rare in the middle ear. Histopathological findings are essential to establish the definitive diagnosis of these tumors. We present the case of a 43-year-old man with a clinical diagnosis of left otitis media with effusion for five years without improvement, with a history of myringotomy with a ventilation tube. He later underwent surgery for a simple mastoidectomy with biopsy. The final diagnosis, based on histopathology and immunohistochemical studies, was a NET of the middle ear. The morphological findings of this type of tumor should be considered based on the differential diagnosis of neoplasias that develop in the middle ear; however, in many cases, the NET in this location may be underdiagnosed. The recommended treatment is surgical resection and long-term follow-up. The main objective of this article is to describe the presentation of NETs in the middle ear, although rare, to help detect more cases through education and personal experience.

## Introduction

Neuroendocrine tumors (NETs) of the middle ear are exceptionally rare neoplasms, accounting for less than 2% of primary tumors of the middle and inner ear, as reported in the 2022 WHO Classification of Head and Neck Tumours [1-3]. A recent systematic review included 198 reported cases, reflecting the limited number of documented cases in the literature [4]. Their nomenclature has varied over time, including middle ear adenoma, carcinoid tumor, adenomatous tumor, adenocarcinoid tumor, amphicrine tumor, and adenomatous NET, reflecting the dual epithelial and neuroendocrine differentiation that characterizes these lesions and has contributed to their historically controversial classification [5].

Unlike neuroendocrine neoplasms of the gastrointestinal tract, pancreas, and lung, middle ear NETs arise in an anatomic site where native neuroendocrine cells are not usually identified, making their histogenesis uncertain. Clinically, they most often affect adults in the fourth to fifth decades of life and typically present with nonspecific otologic symptoms, including conductive or mixed hearing loss, aural fullness, tinnitus, otalgia, or chronic otorrhea [6]. Because these findings overlap with much more common entities such as chronic otitis media, cholesteatoma, paraganglioma, and other middle ear masses, preoperative diagnosis is challenging.

Imaging studies, including computed tomography and magnetic resonance imaging, are useful for assessing tumor extent and guiding surgical planning; however, they are not diagnostic. Definitive diagnosis requires histopathological evaluation supported by immunohistochemistry, demonstrating epithelial differentiation together with diffuse neuroendocrine marker expression [5]. According to the 2022 WHO classification of head and neck neuroendocrine neoplasms, middle ear NETs are classified as well-differentiated epithelial neuroendocrine neoplasms, distinct from poorly differentiated neuroendocrine carcinomas [2,6-8].

Because these tumors may clinically mimic chronic otitis media with effusion or other benign inflammatory middle ear conditions, they may remain unsuspected for years, leading to delayed diagnosis. This is clinically relevant because, despite their often low-grade morphology, middle ear NETs may show unpredictable biological behavior. In published case-based reviews and recent literature, reported local recurrence rates range from 12.7% to approximately 25%, while local or distant metastatic disease has been described in 4% to 9% of cases, sometimes occurring many years after initial treatment [4,6]. These findings support the need for long-term surveillance.

This diagnostic pitfall is illustrated by the present case. We report a 43-year-old man with a prolonged history of left-sided otologic symptoms clinically suggestive of chronic otitis media, in whom histopathological and immunohistochemical examination established the diagnosis of a middle ear NET.

## Case presentation

A 43-year-old man presented with a five-year history of left-sided aural fullness and hearing loss, initially managed as otitis media with effusion. Initial management included antimicrobial therapy and myringotomy with ventilation tube placement, without clinical improvement. His medical history was notable for occupational use of synthetic earplugs for approximately 10 years before symptom onset. The patient reported left-sided aural fullness and hearing loss, without otalgia or otorrhea. Physical examination showed normally positioned auricles. Left otoscopy revealed a patent external auditory canal and a diffusely erythematous, inflamed tympanic membrane, without otorrhea. Right otoscopy was unremarkable. Pure-tone audiometry demonstrated mild conductive hearing loss, with an air-bone gap of approximately 15-20 dB at mid-frequencies. Speech discrimination was preserved (>90%). Tympanometry showed a type A curve, consistent with preserved mobility of the tympano-ossicular system.

Given the persistence and insidious progression of symptoms, computed tomography of the temporal bones was performed. Imaging showed a well-developed and well-pneumatized left mastoid, with soft-tissue opacification involving the epitympanum, mesotympanum, and hypotympanum, without evidence of bone erosion. The tympanic membrane appeared thickened and bulging (Figure 1).

**Figure 1:**
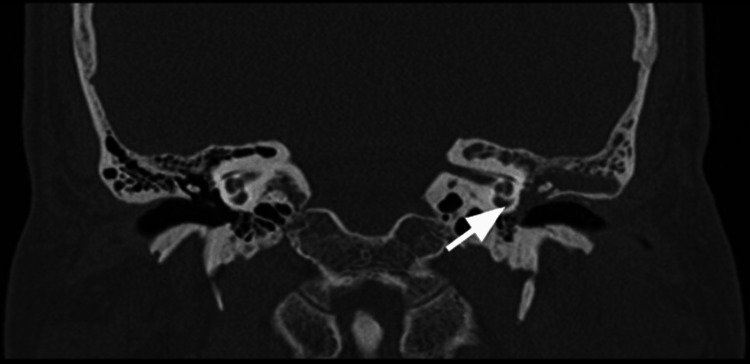
Temporal bone CT Coronal computed tomography image of the temporal bones showing a well-pneumatized left mastoid and soft-tissue opacification involving the left epitympanum, mesotympanum, and hypotympanum, with thickening and bulging of the tympanic membrane (arrow). No bony erosion is identified.

The indication for surgery was not based exclusively on the audiologic findings, but rather on the identification of a localized middle ear lesion. Clinical and imaging studies demonstrated a small, well-circumscribed lesion considered amenable to complete resection. The patient underwent a simple mastoidectomy with exploration of the left middle ear. Intraoperatively, whitish, friable tissue with granular areas was identified. Complete surgical excision was achieved, and no gross residual lesion was identified. The middle ear ossicles were intact, with no evidence of fracture, dislocation, or disruption of the ossicular chain.

The resected tissue was submitted to the Anatomic Pathology Department as “left middle ear mucosa.” Gross examination revealed multiple irregular grayish-brown tissue fragments with reddish areas and a mucoid appearance, measuring 0.8 x 0.5 × 0.2 cm in aggregate. The specimen was entirely submitted for histological examination (Figure 2).

**Figure 2:**
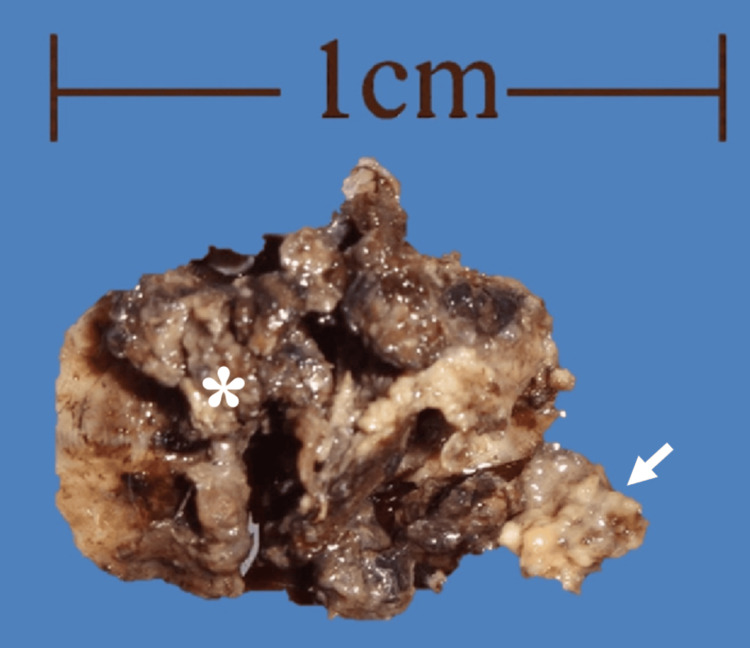
Grossing appearance The specimen consists of multiple irregular, lobulated, reddish-yellow mucoid tissue fragments, measuring 0.8 × 0.5 × 0.2 cm in aggregate. The asterisk (*) indicates the granular-appearing tumor tissue, and the arrow indicates the external auditory canal mucosa.

Hematoxylin and eosin-stained sections showed a neoplastic lesion composed of nodules of round-to-cuboidal cells with hyperchromatic nuclei and regular nuclear contours. The tumor cells were arranged in trabecular and solid sheet-like patterns. No mitotic figures were identified, and the mitotic count was 0 mitoses per 2 mm². Immunohistochemical staining showed diffuse positivity for chromogranin and synaptophysin in the neoplastic cells. The Ki-67 proliferation index was assessed in areas of highest nuclear labeling and was estimated to be less than 2% (Figure 3). Based on the histopathological and immunohistochemical findings, a diagnosis of NET of the left middle ear, WHO grade 1, was established.

**Figure 3:**
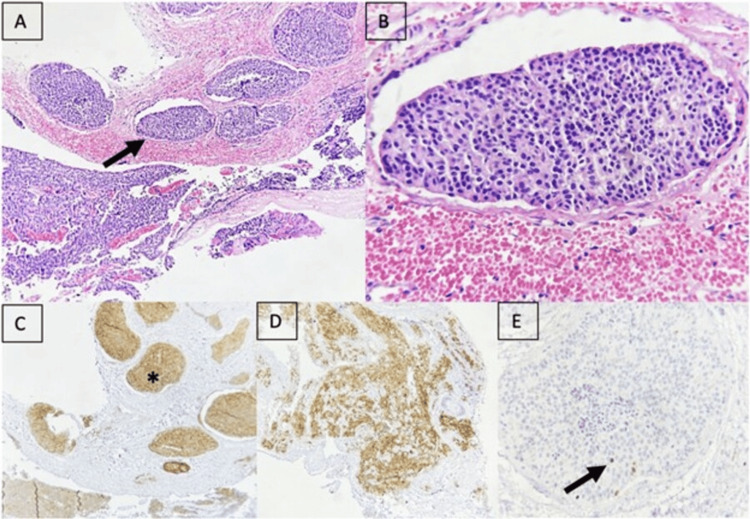
Histology and immunohistochemical findings (A) Neoplastic lesion arranged in nodules and trabeculae of round-to-cuboidal tumor cells within fibroconnective stroma (arrow), hematoxylin and eosin (×4). (B) Higher-magnification view showing a relatively uniform population of round-to-cuboidal tumor cells with no mitotic figures identified in 2 mm², hematoxylin and eosin (×40). (C) Cytoplasmic positivity for chromogranin (asterisk) (×4). (D) Cytoplasmic positivity for synaptophysin (×4). (E) Low Ki-67 proliferation index, less than 2% (arrow) (×40).

Postoperative examination showed adequate surgical recovery, with a patent left external auditory canal and an intact tympanic membrane, without otorrhea. Follow-up demonstrated improvement in hearing loss and partial resolution of aural fullness. Postoperative imaging showed no radiologic evidence of gross residual tumor in the surgical bed. At 24 months postoperatively, the patient remains under clinical surveillance with no evidence of recurrence to date. Long-term follow-up was planned, including otologic evaluation every six months and periodic imaging during the first postoperative years, given the risk of residual disease, late recurrence, or metastatic spread.

## Discussion

Middle ear NETs are extremely rare. The term "adenoma" has been questioned because these tumors may show local invasion and, in rare cases, metastatic behavior [9]. They most commonly occur in the fifth decade of life and affect both sexes equally [10]. The most frequent symptoms include hearing loss (usually conductive), aural fullness, tinnitus, and/or otalgia, whereas facial nerve palsy is rare [11,12]. However, the clinical presentation is nonspecific and may mimic chronic otitis media [13]. Consistent with previous reports, our patient presented with left-sided aural fullness and hearing loss clinically suggestive of chronic otitis media, without facial paralysis or systemic symptoms. A complete otoscopic examination, imaging studies, and histopathological evaluation are therefore crucial for establishing an accurate diagnosis.

According to the 2022 WHO classification of head and neck neuroendocrine neoplasms, the diagnosis of a NET requires appropriate morphology together with diffuse neuroendocrine differentiation demonstrated in virtually all tumor cells. Within this framework, well-differentiated epithelial neuroendocrine neoplasms are classified as NET G1, NET G2, or NET G3. NET G1 is defined by the absence of necrosis, fewer than 2 mitoses per 2 mm², and a Ki-67 index of less than 20%. NET G2 is defined by the presence of necrosis and/or 2-10 mitoses per 2 mm², with a Ki-67 index of less than 20%. NET G3 is defined by more than 10 mitoses per 2 mm² and/or a Ki-67 index greater than 20%, in the absence of poorly differentiated cytomorphology. In contrast, neuroendocrine carcinomas are poorly differentiated neoplasms with more than 10 mitoses per 2 mm² and a Ki-67 index greater than 20%, often exceeding 55%, and are further subclassified as small-cell or large-cell neuroendocrine carcinoma according to cytomorphology [2,14].

The differential diagnosis includes both benign and malignant conditions, such as cholesteatoma, jugulotympanic paraganglioma, vestibular schwannoma, meningioma, and adenocarcinoma [7,9,15-19]. Cholesteatoma is characterized by keratinizing squamous epithelium and laminated keratin debris rather than a true epithelial neuroendocrine proliferation [16]. Vestibular schwannoma shows diffuse S100 and SOX10 positivity and lacks epithelial and neuroendocrine differentiation [17]. Meningioma may express EMA, PR, and SSTR2A and usually shows meningothelial architecture, whorls, or psammoma bodies [18]. Primary middle ear adenocarcinoma is a rare malignant epithelial neoplasm that may exhibit gland-forming architecture but lacks the diffuse neuroendocrine phenotype required for middle ear NET. Focal neuroendocrine marker expression in a carcinoma is insufficient for a diagnosis of NET and should be interpreted in conjunction with morphology and staining extent [7]. Although a jugulotympanic paraganglioma may share diffuse neuroendocrine marker expression with a middle ear NET, it typically shows a classic Zellballen pattern with prominent, delicate vascular stroma. Lack of cytokeratin expression, together with an S100- or SOX10-positive sustentacular cell network, supports the diagnosis of paraganglioma [19]. When morphology alone is not sufficient, immunohistochemistry may help distinguish these entities (Table 1).

**Table 1:** Immunohistochemical differential diagnosis of middle ear neuroendocrine tumor Immunohistochemical profiles are summarized as typical patterns and should be interpreted in conjunction with morphology. *EMA*, epithelial membrane antigen; *PR*, progesterone receptor; *SSTR2A*, somatostatin receptor 2A.

Diagnosis	Synaptophysin / Chromogranin	Cytokeratins	S100 / SOX10	EMA / PR / SSTR2A	Morphology
Middle ear neuroendocrine tumor [2,3,14]	Diffusely positive	Positive	Negative	Usually negative or variable	Well-differentiated epithelial nests, trabeculae, glands, or cords
Paraganglioma [19]	Diffusely positive	Negative	Positive in sustentacular cells	Negative	Zellballen architecture with delicate vascular stroma
Schwannoma [17]	Negative	Negative	Diffusely positive	Negative	Spindle cell neoplasm with Antoni A and Antoni B areas
Meningioma [18]	Negative or rarely focal	Negative or variable	Usually negative	Positive	Whorls, syncytial growth, and psammoma bodies
Adenocarcinoma [7]	Negative or focal	Positive	Negative	Variable	Infiltrative gland-forming epithelial neoplasm
Cholesteatoma [16]	Negative	Positive in squamous epithelium	Negative	Negative	Keratinizing squamous epithelium with laminated keratin debris

Microscopically, middle ear NETs are composed predominantly of cuboidal to columnar cells with pale eosinophilic cytoplasm and round to oval nuclei showing minimal pleomorphism [13]. The chromatin may show a “salt-and-pepper” pattern, supporting neuroendocrine differentiation. Several architectural patterns have been described, including glandular, tubular, solid, trabecular, nested, cribriform, cystic, lamellar, and single-cell growth patterns, and these tumors usually show positivity for neuroendocrine markers and cytokeratins [10]. As in other anatomic sites, confirmation of neuroendocrine differentiation is an essential step in the diagnostic work-up. Immunohistochemical evaluation may include neuroendocrine markers such as INSM1, chromogranin A, and synaptophysin, together with cytokeratins and Ki-67/MIB-1 [2,14]. Strict correlation between morphology and immunohistochemical findings is essential for accurate diagnosis.

On CT, middle ear NETs usually appear as well-demarcated soft-tissue masses without bone erosion. In some cases, they may involve or erode the ossicular chain; however, extensive bone destruction or invasion of adjacent bone is uncommon [9,10]. In our case, CT showed soft-tissue opacification involving the epitympanum, mesotympanum, and hypotympanum, without evidence of bony erosion. Otoscopic examination showed a thickened and bulging tympanic membrane. After surgical resection, no gross residual lesion was identified. These findings are consistent with previously reported cases and support the radiological impression of a non-destructive middle ear mass.

Macroscopically, middle ear NETs are usually non-encapsulated lesions with a soft-to-firm consistency. They may appear white, grayish, or reddish-brown and range from 0.2 to 3.0 cm in greatest dimension [15]. In the present case, fragmented tissue measuring 0.8 cm in greatest dimension was obtained. The fragments were grayish-brown, with reddish areas and a mucoid appearance.

In our case, the tumor showed solid and trabecular architecture and was composed of round-to-cuboidal cells with regular nuclear contours, finely granular chromatin, and scant-to-moderate cytoplasm. No necrosis or mitotic figures were identified, and the mitotic count was 0 mitoses per 2 mm². Immunohistochemistry demonstrated diffuse positivity for synaptophysin and chromogranin. The Ki-67 proliferation index was assessed in areas of highest nuclear labeling and was estimated to be less than 2%. Applying the 2022 WHO criteria, the well-differentiated morphology, absence of necrosis, mitotic count of 0 mitoses per 2 mm², Ki-67 proliferation index of less than 2%, and diffuse neuroendocrine marker expression supported classification as a WHO grade 1 middle ear NET.

Surgical excision remains the mainstay of treatment for middle ear NETs, although the optimal surgical approach should be individualized according to tumor extent, ossicular involvement, and mastoid extension [3]. In this case, gross total resection was performed, and postoperative imaging showed no radiologic evidence of gross residual tumor, with no clinical or radiologic recurrence at 24 months. The absence of recurrence during short- to mid-term follow-up does not exclude the possibility of late recurrence or metastatic spread. Prolonged surveillance is warranted, including periodic otologic evaluation and imaging follow-up, particularly during the first years after surgery.

## Conclusions

Primary tumors of the middle ear are rare, and middle ear NETs are particularly uncommon. These tumors usually present with nonspecific symptoms, and imaging findings are often non-characteristic. Diagnosis cannot rely on clinical or radiological features alone. The present case highlights that in patients with persistent unilateral otitis media with effusion, aural fullness, or hearing loss that does not improve with standard treatment, an underlying middle ear neoplasm may warrant consideration as part of the differential diagnosis. Definitive diagnosis requires histopathological examination supported by immunohistochemical studies. Surgical resection remains the main treatment, and long-term follow-up is advisable to detect residual disease, late recurrence, or metastatic spread.
